# Exploratory ATR-FTIR analysis with spectral deconvolution of polymer-like spectral signatures in suspended matter from the Atoyac River, Mexico

**DOI:** 10.1007/s10661-026-15507-7

**Published:** 2026-05-29

**Authors:** Wendy Argelia García-Suastegui, Orlando Zaca-Morán, Paola Guadalupe Gordillo-Guerra, Ana Cristina Covarrubias-Lopez, Anabella Handal-Silva, Celia Lizeth Gomez, Irma Daniela Silva-Adaya, Julio César Ramírez-San-Juan, Juan Pablo Padilla-Martínez

**Affiliations:** 1 Instituto de Ciencias, Benemérita Universidad Autónoma de Puebla, 72570 Puebla, México; 2 Centro de Investigación en Biotecnología Aplicada, Instituto Politécnico Nacional, 90700 Tepetitla, Tlaxcala México; 3 Departamento de Sistemas Biológicos, Universidad Autónoma Metropolitana, Unidad Xochimilco, 04960 Ciudad de Mexico, México; 4 Laboratorio Experimental de Enfermedades Neurodegenerativas, Instituto Nacional de Neurología y Neurocirugía, 14269 Ciudad de Mexico, México; 5 Coordinación de Óptica, Instituto Nacional de Astrofísica, Óptica y Electrónica, 72840 Tonantzintla, Puebla, México

**Keywords:** ATR-FTIR, Polymer-like spectral features, Suspended matter, Freshwater pollution, Atoyac River

## Abstract

The Atoyac River is one of the most impacted freshwater systems in central Mexico due to sustained industrial, agricultural, and domestic discharges. Despite increasing concern regarding plastic contamination in rivers, information on polymer-related materials in this system remains limited. In this study, a qualitative and exploratory assessment of polymer-like spectral features in suspended matter was conducted along a 43-km section of the Atoyac River. Water samples were collected at six sites representing contrasting land use influences and analyzed directly by ATR-FTIR spectroscopy without chemical or enzymatic removal of organic matter. To improve interpretation of the complex spectra obtained from this highly polluted matrix, Gaussian deconvolution was applied to overlapping absorption regions. Recurrent infrared bands compatible with aliphatic, aromatic, ester, carbonate, and amide-containing structures were detected, showing spectral similarity to common synthetic polymers such as polyethylene-, polypropylene-, polyethylene terephthalate-, polystyrene-, polycarbonate-, and polyamide-like materials. A prominent absorption near 1121 cm⁻^1^, consistent with sulfonated functional groups commonly associated with textile dyes and other industrial sulfonated compounds, was observed at sites influenced by textile activity. Given the absence of organic matter digestion, the results are interpreted as indicative of polymer-like spectral signatures rather than definitive microplastic identification. This work demonstrates the potential of ATR-FTIR combined with spectral deconvolution as a rapid screening approach for polymer-related contamination in complex freshwater environments and provides a qualitative baseline for future studies employing standardized microplastic extraction and quantification protocols.

## Introduction

Surface water pollution is a major global environmental concern, with rivers acting as primary pathways for the transport of contaminants derived from anthropogenic activities. Among these pollutants, plastic-derived materials have attracted increasing attention due to the persistence and widespread distribution of synthetic polymers in aquatic environments. Environmental degradation processes such as ultraviolet radiation, mechanical abrasion, and biological activity fragment plastic debris into progressively smaller particles, increasing their environmental mobility and potential ecological impact (Fu et al., 2020).

The Atoyac River, located in central Mexico and flowing through the states of Puebla and Tlaxcala, exemplifies the environmental pressures experienced by urban rivers in developing regions. Intensive industrial activity, agricultural runoff, and insufficient wastewater treatment have resulted in severe degradation of water quality (Covarrubias-López et al., 2023; Estrada-Rivera et al., 2022), with documented alterations in physicochemical parameters and high levels of toxic pollutants (Pérez-Castresana et al., 2018). Despite supporting the livelihoods of more than three million people (INEGI, 2020), the occurrence and spatial distribution of synthetic polymers in the river remain largely unexplored.

Synthetic polymers such as polyethylene (PE), polyethylene terephthalate (PET), polyvinyl chloride (PVC), polypropylene (PP), and polystyrene (PS) are widely used in industrial and consumer applications (Veerasingam et al., 2021). Once fragmented into microplastics—commonly defined as plastic particles smaller than 5 mm, although the lower size limit remains dependent on analytical constraints and study design (GESAMP, 2015)—these materials may pose enhanced environmental risks due to their capacity to sorb toxic additives and persistent organic pollutants (Huerta-Lwanga 2016). Experimental studies have shown adverse effects on aquatic organisms, including inhibited algal growth and disrupted primary productivity (Feng et al., 2020; Yang et al., 2020), as well as potential human health risks associated with plastic additives such as phthalates and bisphenol A (Chen et al., 2017; Heudorf et al., 2007; Hlisníková et al., 2021; Liu et al., 2019; Panagiotou et al., 2021). However, the identification of polymer-related materials in highly polluted rivers like the Atoyac is analytically challenging due to the abundance of natural organic matter and suspended solids, which produce complex, overlapping infrared signals. Conventional digestion-based protocols may not be feasible in exploratory settings, underscoring the need for rapid screening methods applicable to untreated samples.

Fourier transform infrared (FTIR) spectroscopy is one of the most widely used analytical techniques for polymer characterization, owing to its ability to identify functional groups and polymer backbones. However, in complex environmental matrices such as untreated river water, spectral overlap between synthetic polymers and natural organic matter presents a major analytical challenge. Organic compounds commonly present in suspended matter exhibit absorption bands that can partially or completely overlap with characteristic polymer vibrations, complicating unambiguous identification. This limitation is further exacerbated when samples are analyzed without prior chemical treatment, where matrix interferences are more pronounced.

To address these challenges, mathematical spectral deconvolution techniques have been proposed as an alternative approach to enhance spectral resolution by resolving overlapping bands into individual component peaks, thereby improving interpretability (Laurson et al., 2020). These methods, including Fourier self-deconvolution and derivative-based approaches, have been successfully applied in different spectroscopic contexts to extract hidden spectral information and improve band assignment (Barnés-Calle et al., 2026; Laurson et al., 2020; Ortuso et al., 2019). For instance, FTIR deconvolution has been used to resolve overlapping bands in complex biological systems, such as protein secondary structure analysis, although its sensitivity to methodological parameters has also been emphasized (Barnés-Calle et al., 2026). Despite these advances, the application of spectral deconvolution for the identification of polymeric signatures in complex environmental samples remains limited.

Building upon these considerations, this study employs a combined exploratory ATR-FTIR and Gaussian spectral deconvolution approach to investigate polymer-like spectral signatures in suspended matter from the Atoyac River. The analysis focuses on untreated water samples (absence of digestion) from highly impacted river sections, aiming to identify reproducible polymer-compatible spectral features rather than definitive microplastic quantification. The results provide a qualitative baseline for future studies employing standardized digestion and separation protocols and contribute to the development of improved monitoring strategies for plastic-derived pollution in polluted freshwater systems.

## Materials and methods

### Study area

**Fig. 1:**
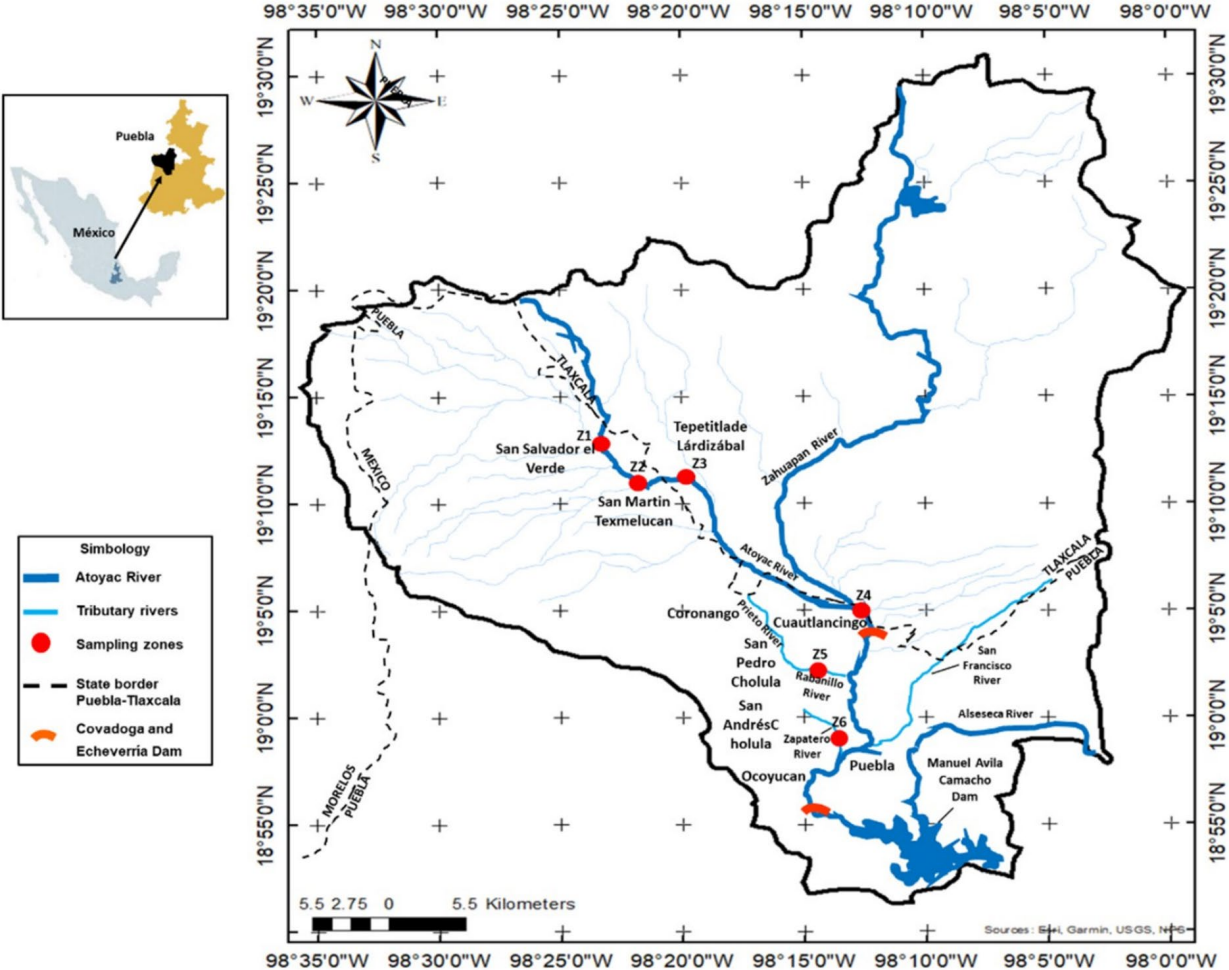
Location of the sampling municipalities. These are in the Atoyac River and its tributaries in Tlaxcala and Puebla, Mexico

The Atoyac River is part of the Alto Atoyac Basin, located in the Tlaxcala-Puebla Valley in the IV Balsas Hydrological-Administrative Region, as shown in Fig. 1 (CONAGUA 2018; DOF 2010; INEGI, 2010). It is formed by the melting of the Iztaccíhuatl volcano and is the longest watercourse in the state of Puebla. With a length of almost 200 km, it has an approximate area of 4130 km^2^ and approximately 3.3 million inhabitants live in its vicinity (Martinez-Tavera et al., 2017). Downstream, it crosses the towns of San Salvador El Verde and San Martín Texmelucan de Labastida in the state of Puebla for about 30 km before entering the state of Tlaxcala at the town of Tepetitla de Lardizábal, where it flows for 29 km before returning to Puebla for 25 km until it enters the Manuel Ávila Camacho Dam. Its tributaries, the Rabanillo and Zapatero Rivers, cross the metropolitan area of Puebla. The region has a subhumid temperate climate with summer rains, with an average temperature of 16.9 °C and an average annual rainfall of 827 mm (Pérez Castresana et al. 2018; CONAGUA 2018). Land use in the region is heterogeneous: 64% of the land is dedicated to agricultural production, 22% to forests, 5% to pastures, and about 9% to urban areas with residential and industrial activities (Bravo-Inclán et al., 2015). The main industries in the region are textiles, leather, chemicals, plastics, food, and paper.

### Sample collection

The section of the river system studied (approximately 43 km) begins in the municipality of San Salvador El Verde and ends in the municipality of San Andrés Cholula (see Fig. 1). Water samples were collected during the dry season, when hydrological conditions were relatively stable, to minimize variability associated with rainfall and flow fluctuations. Clean and noncontaminating sampling equipment was required to avoid the introduction of external microplastics. Top-surface water was collected to cover the floating microplastics. Each sample was obtained in triplicate to ensure representativeness and then placed in 2-L glass containers that had been cleaned and rinsed to avoid contamination. Samples were collected directly from the river at six different locations along this section of the river system in accordance with the Mexican Standard for Surface Sampling in Receivers (NMX-AA-14–1980) and were immediately stored under refrigeration at approximately 4 °C in the dark. To preserve their physicochemical integrity, all samples were analyzed within a maximum period of 2 weeks after collection. The municipalities of interest (five municipalities in Puebla and one in Tlaxcala) were San Salvador El Verde (SSV), San Martín Texmelucan de Labastida (SMTL), Tepetitla de Lardizábal (TL), Cuautlancingo (C), San Pedro Cholula (SPC), and San Andrés Cholula (SAC). The geographic coordinates of the sampling sites are shown in Table 1.

**Table 1:** Geographic coordinates of sampling sites

Zone	Municipality	Coordinates	Zone	Municipality	Coordinates
1	SSV	19° 19′ 22″ N 98° 27′ 40″ W	4	C	19° 09′ 06″ N 98° 13′ 44″ W
2	SMTL	19° 16′ 59.1″ N 98° 25′ 31.4″ W	5	SPC	19° 04′ 44″ N 98° 16′ 25″ W
3	TL	19° 17′ 25.8″ N 98° 18′ 1.6″ W	6	SAC	19° 03′ 03″ N 98° 16′ 41″ W

### ATR-FTIR analysis of suspended matter

To remove large debris, water samples were passed through a 5-mm stainless sieve. The samples were vortexed for 30 s using a Vortex-Genie 2 to homogenize the suspended material. A 5-µL aliquot was then deposited directly onto the diamond/ZnSe crystal of a BRUKER Vertex 70 FTIR spectrometer operating in attenuated total reflection (ATR) mode and was allowed to evaporate at room temperature (23 °C), promoting the concentration of suspended solids on the crystal surface. Prior to analysis, samples were homogenized to improve the representativeness of the deposited aliquot within the constraints of ATR-FTIR thin-film requirements.

No strict lower particle size cutoff was imposed; therefore, the analyzed material comprises a broad distribution of suspended particles, ranging from visible fragments to fine particulate matter. However, it should be noted that ATR-FTIR measurements rely on effective contact between the sample and the crystal surface and the penetration depth of the evanescent wave (typically on the order of 1–2 µm). As a result, the technique may preferentially detect larger particles or aggregated material, while finer particles may be underrepresented in the acquired spectra. Consequently, the obtained spectral data are interpreted as representing the bulk chemical signature of the material in contact with the crystal, rather than the selective identification of individual particles within a defined size fraction.

Spectra were acquired over the range 600–3700 cm⁻^1^ with a resolution of 4 cm⁻^1^ and 120 scans per sample using OPUS 6 software. Background spectra were collected prior to each measurement session under ambient laboratory conditions to ensure proper baseline correction. Baseline correction, normalization, and Gaussian deconvolution were carried out using Origin 6.1. The ATR crystal was carefully cleaned with ethanol and a lint-free tissue between measurements to prevent cross-contamination. Although replicate measurements on the same aliquot were not systematically performed, the study design incorporated spatial replication by analyzing six independent samples collected from different sites along the river, thereby capturing the inherent environmental heterogeneity of the system.

### Spectral deconvolution

Gaussian deconvolution was applied to overlapping spectral regions to enhance band resolution and facilitate interpretation: Region 1 (1000–1200 cm⁻^1^), which contains C–O stretching vibrations characteristic of esters and ethers, and Region 2 (1200–1600 cm⁻^1^), which encompasses C–H bending modes and aromatic ring vibrations. Region 3 (1600–1750 cm⁻^1^), corresponding to C = O stretching vibrations of amides, esters, and ketones, was analyzed separately as it lies outside the classical fingerprint region. This segmentation was applied to reduce the number of fitted Gaussian functions per region, thereby improving fitting stability and minimizing the risk of overparameterization. Additionally, the high-wavenumber region (2800–3600 cm⁻^1^), corresponding to C–H and O–H stretching vibrations, was analyzed separately (Region 4). Individual deconvoluted peaks from this region are summarized in Table 2 rather than fully displayed. Between 3 and 10 Gaussian functions were fitted depending on spectral complexity, with adjusted *R*^2^ values exceeding 0.999. Peak positions were compared with literature-reported absorption bands of common synthetic polymers using a tolerance of ± 5 cm⁻^1^.

**Table 2:** Absorption bands and center values of the Gaussian functions identified in each municipality

Municipality	600–1000 (cm^−1^)	Region 1 (cm^−1^)	Region 2 (cm^−1^)	Region 3 (cm^−1^)	Region 4 (cm^−1^)
SSV	694	1024, 1043, 1075, 1080, 1147	1328, 1360, 1391, 1421, 1451, 1490	1640, 1654, 1664, 1713, 1743	3058, 3075, 3231, 3296, 3336, 3412, 3463, 3518
SMTL	625, 698, 836, 868	1011, 1050, 1086, 1104, 1124, 1134, 1155	1273, 1313, 1350, 1375, 1398, 1418, 1441, 1466, 1511	1629, 1656, 1682, 1688, 1713, 1768	2863, 2916, 2986, 2966, 3061, 3119, 3189, 3254, 3308, 3411
TL	618, 680–685, 836, 868, 993	994, 1065, 1074, 1097, 1115, 1131, 1155, 1165	1307, 1328, 1370, 1408, 1452, 1516	1642, 1653, 1688, 1713	2848, 2916, 2965, 3020, 3077, 3129, 3165, 3216, 3266, 3332, 3405, 3496
C	612, 623, 692, 698, 836	1081, 1089, 1103, 1124, 1149	1267, 1291, 1312, 1335, 1362, 1395, 1396	1634, 1661, 1685, 1713, 1768, 1793	2814, 2902, 2949, 3007, 3050, 3094, 3152, 3203, 3257, 3340, 3424, 3522
SPC	612, 623, 698, 836, 868	987, 1033, 1076, 1086, 1119, 1151, 1157	1276, 1318, 1363, 1400, 1433, 1458, 1514	1610, 1638, 1667, 1694, 1713, 1743, 1768	2896, 3048, 3116, 3191, 3342, 3495, 3502
SAC	612, 692, 836, 868	1023, 1074, 1093, 1120, 1152, 1168	1285, 1333, 1367, 1390, 1413, 1439, 1475, 1522	1640, 1651, 1660, 1676, 1699, 1713	2836, 2902, 2939, 3008, 3077, 3108, 3173, 3225, 3288, 3356, 3389, 3455

### Limitations of sample preparation and analytical scope

No chemical or enzymatic digestion was performed to remove organic matter prior to ATR-FTIR analysis. This was an intentional decision, in line with the exploratory objective of evaluating direct ATR-FTIR analysis with spectral deconvolution as a rapid, minimally invasive screening approach in a highly polluted river system impacted by industrial discharges, elevated organic load, and diverse anthropogenic contaminants. Conventional microplastic analysis workflows often rely on digestion protocols, for example, KOH, H_2_O_2_, or enzymatic treatments to remove organic matter and isolate particles (Mallek & Barceló, 2025; Pfeiffer & Fischer, 2020). However, these procedures may alter the physicochemical properties of polymers and affect surface chemistry (Pfeiffer & Fischer, 2020). In particular, oxidative treatments such as wet peroxide oxidation or Fenton-based reactions, although effective in removing organic matrices, have been associated with partial degradation of microplastics and reduced recovery efficiencies, especially under harsh reaction conditions (Pfeiffer & Fischer, 2020).

By contrast, the approach adopted here preserves the sample’s native chemical complexity, enabling polymer-like spectral features to be detected directly within the environmental matrix. This is particularly relevant for exploratory analyses of heterogeneous systems, such as riverine suspended matter. However, the inability to remove organic matter inherently limits the ability to distinguish synthetic polymers unequivocally from natural or anthropogenic organic compounds with overlapping infrared absorption bands.

Consequently, the results are interpreted as polymer-like spectral signatures rather than as definitive identification of polymers. In this context, the proposed methodology should be considered a supplementary tool within a multistep analytical framework. It provides rapid preliminary screening to guide subsequent, more specific analyses using well-established digestion-based and high-resolution techniques.

## Results

### ATR-FTIR spectral characteristics

**Fig. 2:**
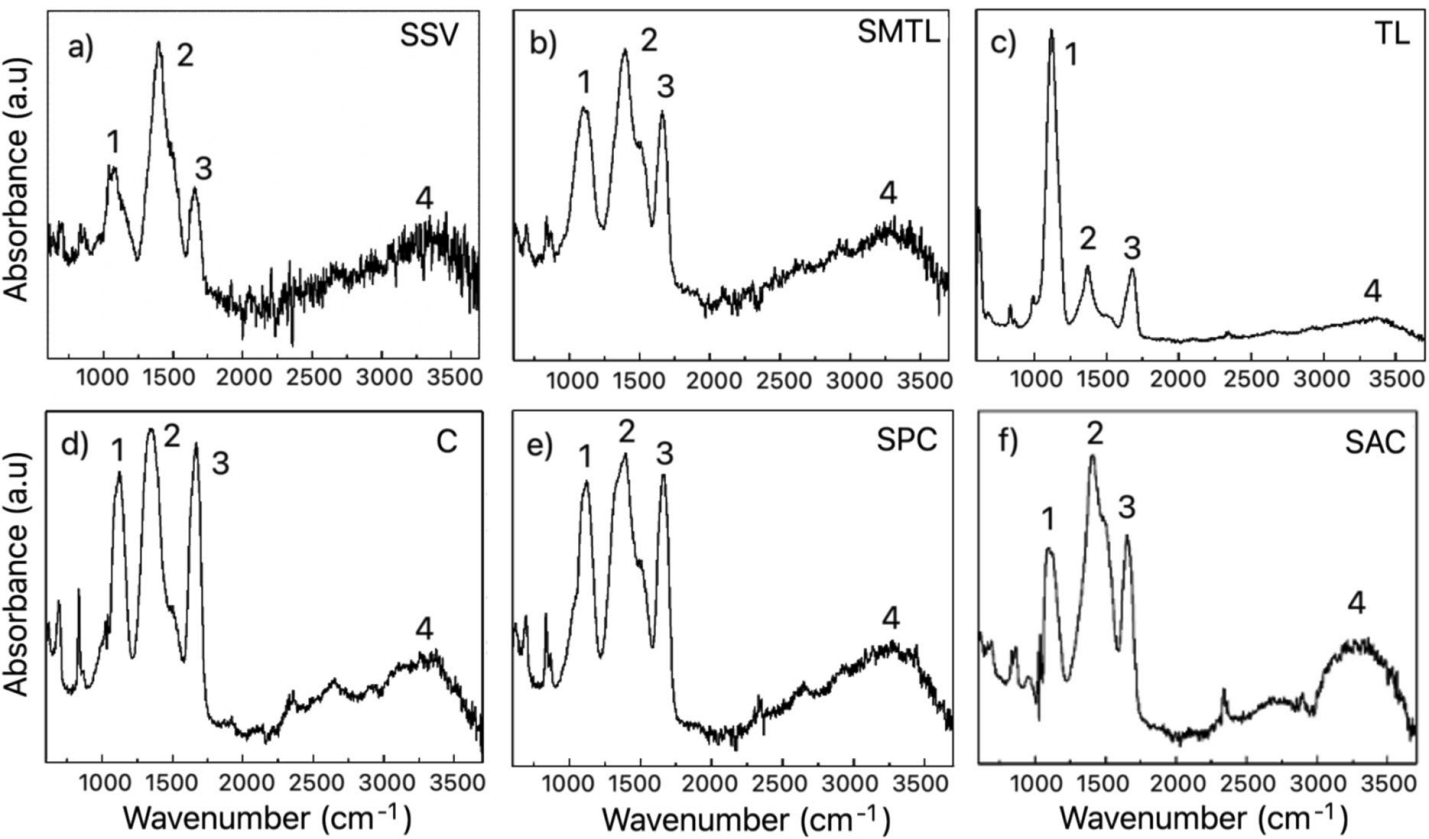
ATR-FTIR spectra (from 600 to 3600 cm^−1^) of water samples from the Atoyac River and its tributaries. **a** San Salvador El Verde (SSV), **b** San Martín Texmelucan de Labastida (SMTL), **c** Tepetitla de Lardizábal (TL), **d** Cuautlancingo (C), **e** San Pedro Cholula (SPC), and **f** San Andrés Cholula (SAC)

The ATR-FTIR absorption spectra of water samples collected at six sites along the Atoyac River and its tributaries exhibited complex and highly overlapped absorption profiles over the 600–3600 cm⁻^1^ range, reflecting the heterogeneous composition of suspended matter in this highly polluted river system (see Fig. 2). Extensive band overlap hindered direct spectral interpretation and polymer characterization. To address this limitation, Gaussian deconvolution analysis was applied to four selected spectral regions characterized by high band density (1000–1200, 1200–1600, 1600–1750, and 2800–3600 cm⁻^1^), as shown in Fig. 2. This approach enabled the separation of overlapping absorptions into individual components, facilitating the qualitative assessment of reproducible polymer-compatible spectral features across sampling sites.

### Deconvolution of overlapping spectral regions

**Fig. 3:**
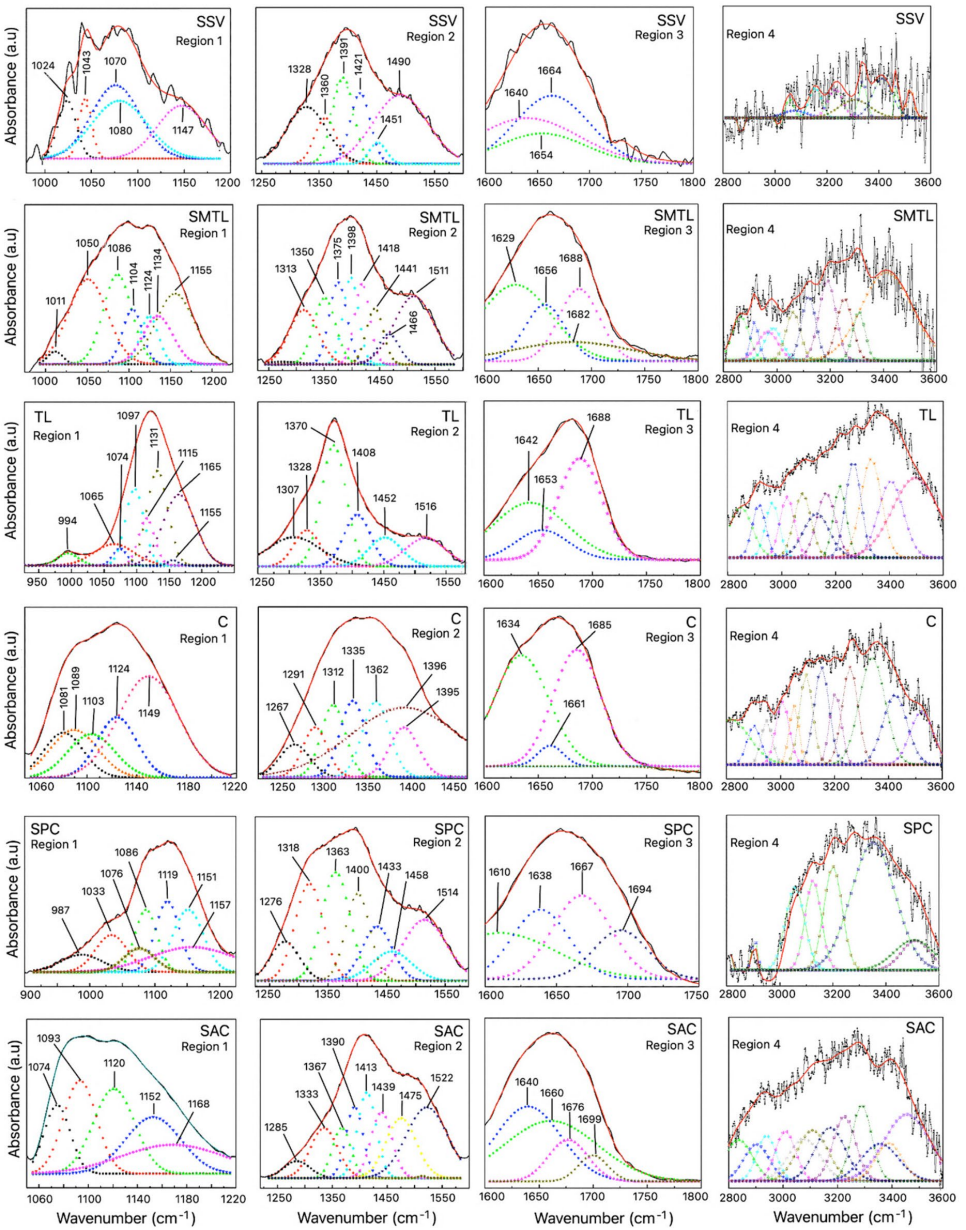
Deconvolution of FTIR spectra in four regions: Region 1 (~ 1000 to 1200 cm^−1^), Region 2 (~ 1200 to 1600 cm^−1^), Region 3 (~ 1600 to 1750 cm^−1^), and Region 4 (2800 to 3600 cm^−1^). To preserve visual clarity, the center values of the Gaussian peaks in Region 4 are not shown here but are shown in Table 2

Gaussian deconvolution was performed on four selected spectral regions of the ATR-FTIR spectra, as shown in Fig. 3. Table 2 summarizes the central wavenumbers of the fitted Gaussian functions and the absorption bands detected in the fingerprint region (600–1000 cm⁻^1^) for each municipality. Gaussian deconvolution enabled the resolution of individual band components within each spectral region, and the resulting peak centers exhibited consistent patterns across sampling sites, indicating the recurrent occurrence of functional group–related spectral features. Across multiple locations, absorption bands compatible with aliphatic C–H stretching, aromatic ring vibrations, carbonyl-containing groups, and amide-associated modes were observed, supporting a qualitative comparison of polymer-compatible spectral features within the highly heterogeneous river matrix. As detailed in the “” section, the results are interpreted as polymer-like spectral signatures rather than definitive polymer identification due to the absence of organic matter digestion.

### Polymer-like spectral features

Gaussian deconvolution enabled the resolution of individual band components within each spectral region, revealing recurrent absorption features across multiple locations. The resulting peak centers showed consistent patterns among sampling sites, suggesting the repeated occurrence of specific functional group–related spectral features within the suspended matter. Major absorption bands were detected in regions commonly associated with C–O, C–H, C = O, aromatic, and amide-related vibrations.

Comparative interpretation of these deconvoluted bands (Table 2) with literature-reported infrared assignments (Table 3) indicated the presence of polymer-compatible spectral features at several sites. Recurrent absorptions in the 1713–1750 cm⁻^1^ region (Jung et al., 2018), typically attributed to ester carbonyl stretching vibrations, were observed at multiple locations. These bands were accompanied by absorptions in the 1290–1340 cm⁻^1^ and 1410–1470 cm⁻^1^ ranges, corresponding to C–O stretching and C–H bending or aromatic-related vibrations, respectively. Such combinations of bands are commonly reported for ester-containing polymeric materials and were consistently detected in several municipalities (Chércoles-Asensio et al. 2009).

**Table 3:**
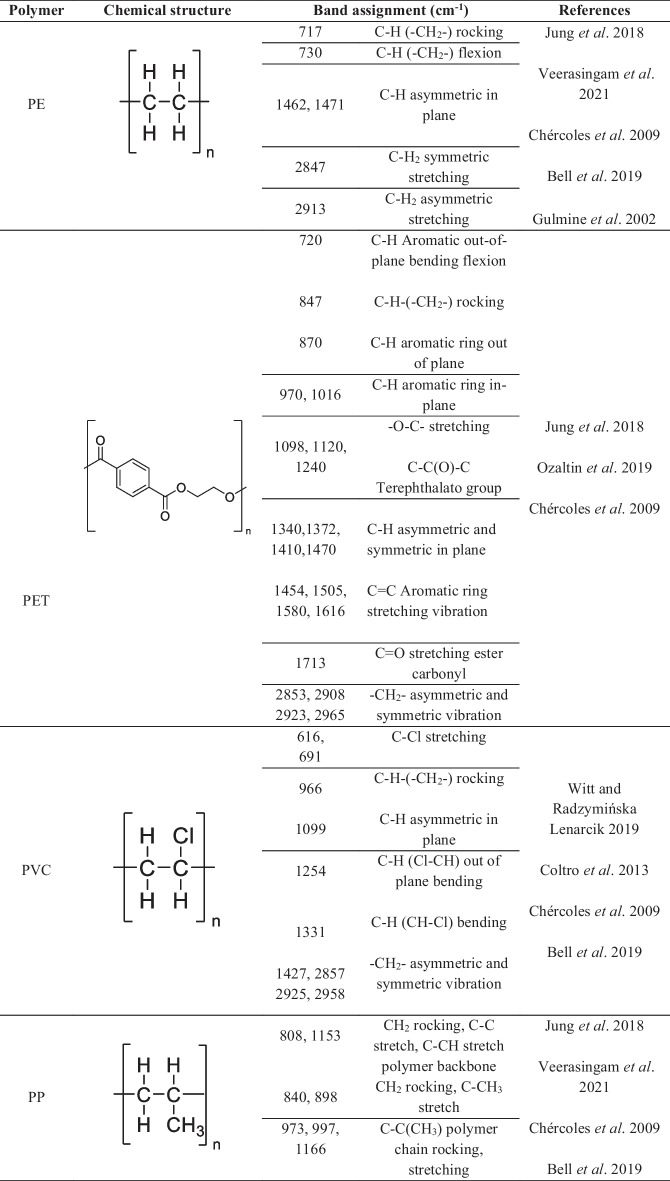

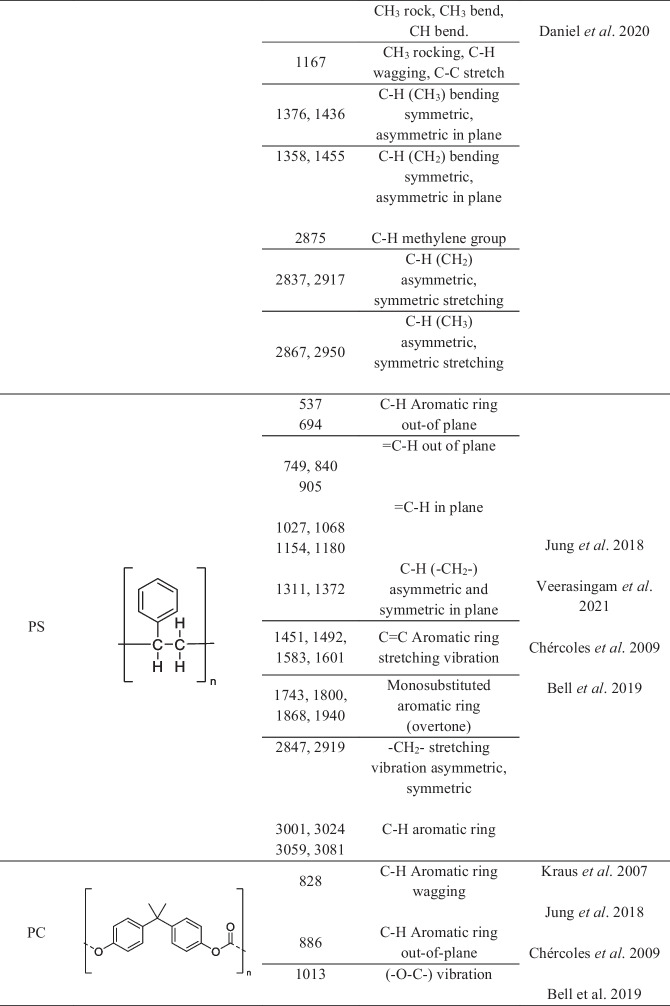

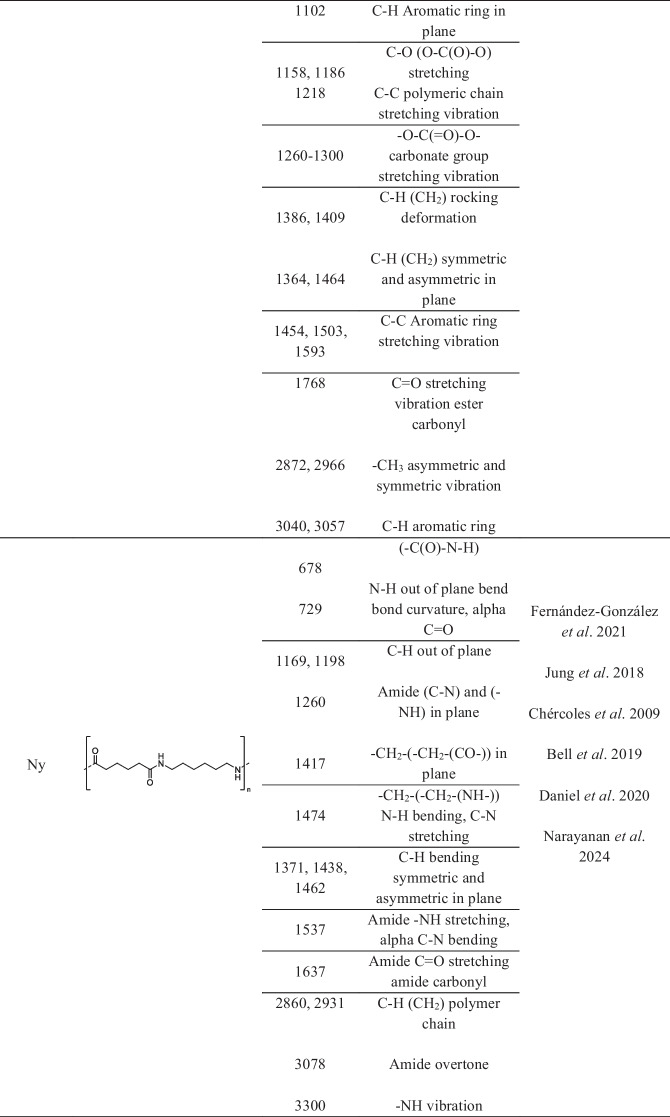
Functional group–related infrared absorption bands and corresponding polymeric materials reported in the literature, used for comparative interpretation of deconvoluted ATRFTIR spectra

Additional carbonyl-related absorptions centered near 1760–1770 cm⁻^1^ were resolved at selected sampling sites. These features, together with supporting absorptions in the 1200–1300 cm⁻^1^ region and aromatic-associated vibrations near 1450–1500 cm⁻^1^, are compatible with carbonate-containing polymer-like materials reported in the literature (Kraus et al., 2007). Although these signals were not uniformly present across all sites, their occurrence at specific locations suggests spatial variability in the distribution of polymer-related spectral features along the river.

Bands in the low-wavenumber region between 616 and 691 cm⁻^1^, commonly associated with C–Cl stretching vibrations, were detected at most sampling points, together with absorptions near 1250–1330 cm⁻^1^ and characteristic CH_2_ stretching bands in the 2800–3000 cm⁻^1^ region. These spectral features are compatible with functional groups reported for chlorinated polymeric materials and were absent or less pronounced at selected upstream locations (Bell et al., 2019; Witt & Radzymińska-Lenarcik, 2019).

Polymer-compatible absorptions related to aliphatic hydrocarbon chains were also recurrently observed across multiple sites. These included CH_2_ and CH_3_ bending modes near 1370–1470 cm⁻^1^ and symmetric and asymmetric CH_2_ stretching vibrations between 2840 and 2950 cm⁻^1^. Such features are widely reported for saturated hydrocarbon-based polymers and were among the most consistently detected spectral components within the suspended matter (Chércoles-Asensio et al., 2009; Daniel et al., 2020).

At selected sites, absorptions associated with aromatic structures were resolved, including bands in the 690–700 cm⁻^1^ region corresponding to aromatic C–H out-of-plane bending (Bell et al., 2019) and strong absorptions between 1450 and 1600 cm⁻^1^ attributable to aromatic ring vibrations (Jung et al., 2018; Veerasingam et al., 2021). Additional aromatic C–H stretching modes were observed in the 3000–3100 cm⁻^1^ region (Jung et al., 2018). These features are compatible with aromatic-containing polymer-like materials such as polystyrene.

Finally, absorptions consistent with amide-containing structures were detected at several sampling locations, including bands near 1637–1650 cm⁻^1^ (amide I) and 1537–1540 cm⁻^1^ (amide II), accompanied by CH_2_ stretching vibrations between 2860 and 2930 cm⁻^1^ (Fernández-González et al., 2021; Jung et al., 2018). While such spectral features may originate from both natural organic matter (proteins and humic substances) and synthetic polyamides, their recurrent detection across multiple sites confirms the widespread presence of amide-containing compounds within the suspended matter. However, due to the absence of organic matter digestion, these absorptions cannot be unequivocally attributed to synthetic polyamides.

The FTIR bands identified revealed spectral features consistent with those of common synthetic polymers. These features included polyethylene- and polypropylene-like aliphatic chains, ester-containing materials consistent with PET-like structures, aromatic polymers compatible with polystyrene-like materials, carbonate-associated bands suggestive of polycarbonate-like structures, and amide-containing materials consistent with polyamide-like substances. As shown in Table 4, these features were heterogeneously distributed along the river, with greater spectral complexity observed in urban and industrial zones.

**Table 4:** Occurrence of polymer-compatible spectral features inferred from ATR-FTIR functional group analysis across sampling sites. The symbols indicate the presence of infrared absorption bands compatible with functional groups commonly reported for the indicated polymeric materials. These assignments are based on comparative spectral interpretation and do not constitute definitive polymer identification

Zone	Municipality	Polymer-like spectral features						
		PE-like	PET-like	PP-like	PVC-like	PC-like	PS-like	Amide-like
1	San Salvador El Verde (SSV)	-	-	-	-	-	-	√
2	San Martín Texmelucan de Labastida (SMTL)	√	√	√	√	√	√	√
3	Tepetitla de Lardizábal (TL)	√	√	√	√	-	-	√
4	Cuautlancingo (C)	-	√	-	√	-	√	-
5	San Pedro Cholula (SPC)	√	√	√	√	√	-	√
6	San Andrés Cholula (SAC)	√	√	√	√	-	-	√

Amide-like features indicate the presence of infrared absorptions compatible with amide functional groups, which may originate from either synthetic polyamides or natural organic matter

### Sulfonated functional group signal

**Fig. 4:**
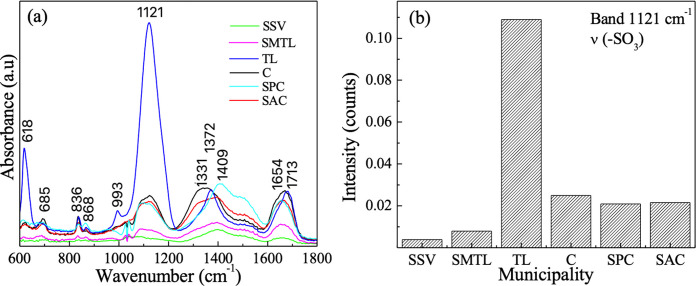
**a** ATR-FTIR absorption spectra and **b** absorption intensity associated with the 1121 cm^−1^ band, both corresponding to the samples of water collected from the Atoyac River and its tributaries

A pronounced absorption near 1121 cm⁻^1^ was observed at sites influenced by textile activity, particularly in industrial areas (Fig. 4). This band is consistent with sulfonated functional groups commonly associated with textile dyes and dye residues (Juárez‐Hernández et al., 2008), indicating a strong industrial contribution to suspended matter composition.

## Discussion

The present study provides a qualitative spectroscopic assessment of suspended matter in untreated water samples from the Atoyac River using ATR-FTIR spectroscopy combined with Gaussian deconvolution. Rather than aiming at definitive identification or quantification of microplastic particles, this work was designed to evaluate whether reproducible polymer-compatible infrared spectral features can be detected within a highly heterogeneous and organic-rich river matrix. This approach responds directly to the analytical challenges posed by heavily polluted freshwater systems, where extensive band overlap and the presence of natural organic matter complicate conventional microplastic characterization (Veerasingam et al., 2021).

The application of Gaussian deconvolution proved effective in resolving overlapping absorption bands and revealing recurrent spectral features across multiple sampling sites. The consistency of peak positions within selected spectral regions suggests that specific functional group–related absorptions are repeatedly present in suspended matter along the river continuum. Similar deconvolution-based approaches have been shown to enhance qualitative interpretation of complex FTIR spectra in environmental matrices, particularly where direct particle isolation or digestion is not feasible (Barnés-Calle et al., 2026; Laurson et al., 2020; Ortuso et al., 2019).

The recurrent detection of ester carbonyl-, aliphatic hydrocarbon-, aromatic-, chlorinated-, and amide-related absorptions across several municipalities suggested the possible widespread occurrence of polymer-compatible spectral features within the Atoyac River system. Interpretation of these polymer-compatible spectral features was supported by comparison with literature-reported functional group assignments, as summarized in Tables 3 and 4. These functional group patterns are consistent with infrared signatures commonly reported for widely used synthetic polymeric materials, including ester-containing, aliphatic, aromatic, and polyamide-based polymers (Coltro et al., 2013; Gulmine et al., 2002; Narayanan and Janardhanan, 2024; Ozaltin et al., 2019; Jung et al., 2018; Chércoles-Asensio et al., 2009). However, it is important to emphasize that such spectral similarities do not constitute definitive evidence of individual microplastic particles, as several of these absorptions may also arise from natural organic matter or mixed anthropogenic inputs.

Spatial variability in the occurrence and intensity of specific spectral features was observed along the river. In particular, the detection of sulfonated-related absorptions at sites influenced by textile activity suggests that local industrial discharges may contribute to the chemical fingerprint of suspended matter. This observation is consistent with previous reports linking sulfonated functional groups to textile processing chemicals and synthetic fibers, particularly sulfonated azo dyes commonly used in textile industry (Khandare et al., 2011). While causal attribution cannot be established within the scope of this study, the spatial correspondence between industrial activity and distinctive spectral features highlights the sensitivity of ATR-FTIR as a screening tool for detecting compositional differences in polluted river systems.

Amide-related absorptions were detected at multiple sampling sites, indicating contributions from amide-containing materials within the suspended matter. Given the known overlap between spectral features of synthetic polyamides and natural proteinaceous compounds (Fernández-González et al., 2021), these signals should be interpreted cautiously. Nevertheless, their recurrent detection across spatially distinct locations raises the possibility that amide-containing materials, whether synthetic or natural, contribute notably to the polymer-compatible spectral background in the river, potentially linked to domestic wastewater inputs and textile-derived fibers.

The qualitative nature of the present results reflects inherent methodological limitations. The absence of chemical digestion or density separation precludes definitive discrimination between synthetic polymers and natural organic constituents, and the use of ATR-FTIR on bulk suspended matter limits particle-level characterization. Despite these constraints, the consistent detection of polymer-compatible spectral features across multiple sites provides valuable baseline information for this highly impacted freshwater system. Such baseline data are particularly relevant in regions where access to standardized microplastic extraction protocols or advanced analytical instrumentation remains limited (Covarrubias-López et al., 2023).

A significant observation in this study is the prominent absorption band at 1121 cm⁻^1^, which was particularly evident in the municipalities of Cuautlancingo (C) and San Pedro Cholula (SPC). This suggests industrial activity and could serve as a potential indicator of textile-related pollution (Juárez‐Hernández et al., 2008), although definitive source attribution would require complementary analyses. This highlights an inherent trade-off in the chosen methodological approach. On the one hand, the absence of organic digestion is a clear limitation, as overlapping signals from natural organic matter introduce ambiguity and preclude definitive polymer identification. On the other hand, in this specific context, it allowed the preservation and detection of sulfonated functional groups associated with textile dyes—spectral features that could be partially degraded or masked by aggressive digestion protocols (e.g., Fenton’s reagent or concentrated H_2_O_2_) (Pfeiffer & Fischer, 2020; Tagg et al., 2017). It is worth noting, however, that alternative, less aggressive digestion methods exist, such as enzymatic treatments or dilute H_2_O_2_ protocols, which may remove interfering organic matter while preserving labile spectral signatures. By employing a direct ATR-FTIR approach coupled with Gaussian deconvolution, we were able to resolve these sulfonated functional groups. Nonetheless, future studies should explore the application of mild digestion protocols to balance matrix interference reduction with the preservation of diagnostically relevant spectral features.

The proposed ATR-FTIR approach with Gaussian deconvolution is not intended to replace conventional microplastic analysis workflows but rather to complement them as an initial screening tool. In resource-limited settings where access to digestion reagents, density separation media, or specialized instrumentation (e.g., micro-FTIR, Raman, or pyrolysis-GC/MS) may be constrained, this method offers a rapid, low-cost, and low-preparation alternative for detecting polymer-compatible spectral signatures directly from untreated water samples. It is particularly well suited for exploratory studies aiming to identify potential contamination hotspots or to generate preliminary evidence that can guide targeted sampling and more resource-intensive analyses.

However, the method has clear boundaries that must be explicitly recognized. The absence of organic matter digestion means that spectral overlap with natural organic compounds (e.g., humic substances, proteins, and polysaccharides) remains a significant source of ambiguity, and definitive polymer identification is not achievable. Therefore, this approach is not suitable for studies requiring quantitative microplastic concentrations or unambiguous polymer identification. Moreover, the method cannot provide information on particle size, shape, or count, which are critical parameters in microplastic risk assessment.

To overcome these limitations, the proposed screening approach should be integrated into a multistep analytical workflow. Positive detections of polymer-like spectral features could be followed by targeted sampling and subsequent analyses employing standardized digestion and density separation protocols, followed by micro-FTIR or Raman spectroscopy for particle-level confirmation. Alternatively, complementary techniques such as pyrolysis-GC/MS could be used to verify polymer identity and quantify mass concentrations. By positioning this method as an entry-level screening tool rather than a definitive analytical technique, its utility is maximized while its limitations are explicitly managed.

## Conclusions

This study provides a qualitative spectroscopic baseline for the assessment of polymer-compatible spectral features in suspended matter from the Atoyac River using ATR-FTIR spectroscopy combined with Gaussian deconvolution. Reproducible functional group–related absorptions associated with ester carbonyl, aliphatic, aromatic, chlorinated, and amide-containing structures were detected across multiple sampling sites, suggesting the widespread presence of synthetic or synthetic-associated spectral signatures within this highly impacted freshwater system.

Although these observations do not constitute definitive identification or quantification of microplastic particles, they highlight the influence of anthropogenic activities, including industrial and domestic inputs, on the chemical fingerprint of suspended matter. The detection of distinctive functional group–related absorptions at textile-influenced sites further underscores the potential contribution of industry-specific sources.

Furthermore, the methodology proved sensitive enough to detect nonpolymeric industrial indicators, such as sulfonated groups (1121 cm⁻^1^) linked to textile dyes. This suggests that the proposed exploratory screening not only indicates the presence of polymer-like materials but also captures a broader chemical fingerprint of the river’s anthropogenic load, offering a more holistic view of the pollution matrix than standardized microplastic protocols that prioritize matrix removal over dye preservation.

Overall, this study suggests that ATR-FTIR spectroscopy combined with Gaussian deconvolution may serve as a useful exploratory screening approach for assessing the presence and spatial distribution of polymer-related spectral features in complex riverine environments. The findings underscore the need for future investigations integrating standardized sample pretreatment, particle isolation, and complementary analytical techniques to validate and quantify microplastic contamination in the Atoyac River. By establishing a qualitative spectroscopic framework, the present work provides a preliminary basis for future investigations of synthetic material inputs in heavily polluted freshwater systems and may support the development of more comprehensive monitoring strategies.

## Data Availability

Data availability is not applicable to this article, as no new data were created or analyzed.
